# Characterization of a Clp Protease Gene Regulator and the Reaeration Response in *Mycobacterium tuberculosis*

**DOI:** 10.1371/journal.pone.0011622

**Published:** 2010-07-16

**Authors:** Ashley M. Sherrid, Tige R. Rustad, Gerard A. Cangelosi, David R. Sherman

**Affiliations:** 1 Seattle Biomedical Research Institute, Seattle, Washington, United States of America; 2 Molecular and Cellular Graduate Program, University of Washington, Seattle, Washington, United States of America; 3 Pathobiology Program and Department of Global Health, University of Washington, Seattle, Washington, United States of America; St Georges University of London, United Kingdom

## Abstract

*Mycobacterium tuberculosis* (MTB) enters a non-replicating state when exposed to low oxygen tension, a condition the bacillus encounters in granulomas during infection. Determining how mycobacteria enter and maintain this state is a major focus of research. However, from a public health standpoint the importance of latent TB is its ability to reactivate. The mechanism by which mycobacteria return to a replicating state upon re-exposure to favorable conditions is not understood. In this study, we utilized reaeration from a defined hypoxia model to characterize the adaptive response of MTB following a return to favorable growth conditions. Global transcriptional analysis identified the ∼100 gene Reaeration Response, induced relative to both log-phase and hypoxic MTB. This response includes chaperones and proteases, as well as the transcription factor *Rv2745c,* which we characterize as a Clp protease gene regulator (ClgR) orthologue. During reaeration, genes repressed during hypoxia are also upregulated in a wave of transcription that includes genes crucial to transcription, translation and oxidative phosphorylation and culminates in bacterial replication. In sum, this study defines a new transcriptional response of MTB with potential relevance to disease, and implicates ClgR as a regulator involved in resumption of replication following hypoxia.

## Introduction

A hallmark of infection with *Mycobacterium tuberculosis* (MTB) is the organism's ability to survive for months to decades in an asymptomatic state, before reactivating in a subset of infected individuals to cause frank disease. Roughly 1/3 of all people worldwide are thought to harbor MTB in a clinically latent state, and 2–10% of these individuals will reactivate during their lifetimes [1], [2], [3]. The threat posed by latency and reactivation is emphasized by the markedly higher 5–10% annual risk of TB disease observed among individuals co-infected with HIV and MTB [4], [5], [6].

The environmental cues that MTB recognizes during latency and reactivation are poorly characterized. Of these, however, oxygen tension may be the best understood [7]. Oxygenation and mycobacterial growth rate are intimately linked, both *in vitro* and *in vivo* [8], [9], [10]. Hypoxia is relevant during infections since in animal models with a similar course of disease to that seen in humans, such as the macaque, granuloma oxygen tension is quite low [11], [12], and human granulomas without airway communication are hypoxic [13]. *In vitro* studies show that hypoxic bacilli halt replication, shift to the glyoxylate cycle, and increase nitrate reduction [14], [15]. In this state MTB requires NAD and ATP synthesis, and maintenance of the proton-motive force [16], [17], indicating that the bacteria remain metabolically active despite halted replication. Underscoring these adaptations are two distinct transcriptional responses: the initial hypoxic response controlled by *dosR* [18], [19], [20], and the enduring hypoxic response or EHR [21].

In contrast to the considerable efforts devoted to elucidating the MTB response to hypoxia, the return to favorable growth conditions is poorly studied. Here we exploit a simple culture model to investigate mechanisms by which MTB resumes growth following reaeration. Prior to replication, MTB upregulates a selection of genes indicating reversal of the adaptations employed during long term hypoxia. These genes encode proteins involved in transcription, translation, cell wall modification and oxidative phosphorylation, and were earlier repressed by the transition from log phase to hypoxia. In addition, our data also reveal a subset of genes induced during reaeration relative to both hypoxia and log phase. This transcriptional profile, which we call the Reaeration Response, is enriched in genes involved in protein degradation and refolding. We also demonstrate that the Reaeration Response transcription factor *Rv2745c* directly regulates the Clp proteases.

## Methods

### Bacterial Strains and Growth Conditions

H37Rv (ATCC 27294) was grown at 37°C in Middlebrook 7H9, 0.05% Tween, 0.2% glycerol and ADC (Becton Dickinson). Stocks were expanded from frozen aliquots within two weeks of experiments. Defined hypoxic assays were performed as previously described [20]. For reaeration, we transferred hypoxic cultures into roller bottles (5∶1 head space ratio) and incubated with rolling at 37°C. Bacteria were enumerated by CFU and most-probable number (MPN) analysis [22]. Alternatively, bacteria were pelleted at 2000 xg for 5 minutes, frozen on dry ice and stored at −80°C for RNA extraction.

### RNA Extraction and Purification

RNA was extracted from cell pellets as previously described [20]. After precipitation, RNA was purified using an RNeasy kit (Qiagen) as recommended by the manufacturer.

### Microarray Analysis

Microarray analysis was performed using arrays provided by JCVI/PFGRC under the NIAID contract N01-AI-15447 using published protocols [23]. Arrays were scanned and spots quantified using Genepix 4000B with GenePix 6.0 software. Data were exported to Acuity 4.1, with spots ‘Not Found’ by the GenePix algorithms excluded from downstream analysis. Identical arrays were analyzed using Statistical Analysis of Microarrays (SAM) [24]. Unless otherwise noted, we defined significance as a mean log_2_ fold change of 1 with a false discovery rate (FDR) of <0.5% in a minimum of three arrays. All data is MIAME compliant and has been deposited in the NCBI Gene Expression Omnibus [25]and are accessible through GEO Series accession number GSE21590 (http://www.ncbi.nlm.nih.gov/geo/query/acc.cgi?acc=GSE21590).

### Quantitative Real-Time PCR

cDNA synthesis and real time PCR were performed as described [21], with each sample normalized to *sigA* expression level.

### Western Blot

Lysates were made by washing and resuspending MTB in 50 mM sodium phosphate buffer pH 8.0 300 mM NaCl 10% glycerol with 1X protease inhibitors (Sigma) then shaking with Lysing Matrix B (Qbiogene) in a Fastprep 120 homogenizer (Qbiogene) and filtering (0.2 µm). SDS-PAGE was performed with 5 ug of lysate per lane, transferred to nitrocellulose then blocked, incubated with primary antibody (anti-ClpP and anti-PtpB rabbit antibodies diluted 1∶5000 and 1∶200 respectively), followed by secondary antibody (Licor Goat anti-rabbit IR680 diluted 1∶5000). Membranes were scanned at 700 nm, quantified and normalized using the Licor Odyssey imaging system.

### Rv2745-S Expression and Purification

The Rv2745c coding sequence was PCR amplified and cloned into pET50b (Novagen), using primers designed to encode two mutations V111D and A112D at the C terminus. Protein expression was induced in *E.coli* BL21(DE3) with IPTG for 3 hours, and cells were lysed with Bugbuster (Novagen). Rv2745c was affinity purified using His-Select Ni-NTA agarose (Sigma), eluted with imidazole (Sigma) and dialyzed into HRV3C protease buffer (Novagen). The NusA fusion protein was removed using HRV3C protease (Novagen) overnight at 4°C, then protease and fusion protein were removed by affinity purification.

### Electrophoretic Mobility Shift Assay

Oligos matching promoter regions of *clpP1P2* (CAACGTGACCGTATGACGCTGTAAGCGAACGCGCCGGTTTCAG) and *clpC1* (CGAGCGGCCATCGGTTCGCCGCCAGCGAACGCGGCAAAGTAC) were ordered with and without 5′ Cy5.5 label, along with the reverse complements (MWG Operon). Oligos were annealed by heating to 95°C then cooling 1°/minute to 4°C. Binding reactions were performed with 200 ng protein and 2 ug of MTB lysate in 25 mM sodium phosphate pH 7.5, 150 mM NaCl, 1 mM EDTA, 2.5 mM MgCl_2_, 1 mM DTT, 1 µg/ml BSA, 10% glycerol containing 1 µg dI:dC DNA and competitor DNA as indicated for 15 minutes at room temperature. 200pmol Cy5.5 labeled probe was added and incubated at 37°C for 5 minutes then 4°C for 25 minutes. Samples were electrophoresed in 5% native polyacrylamide gels for 1 hour at 10 V/cm and scanned by Licor Odyssey.

## Results

### The *M. tuberculosis* Reaeration Response

**Figure 1:**
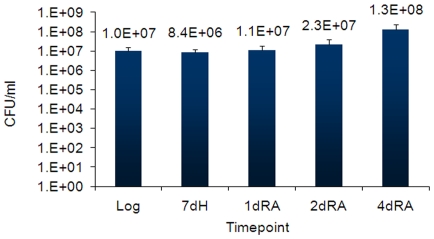
MTB viability and replication during hypoxia and reaeration. CFUs are the average of three independent experiments, each counted in at least triplicate. Error bars are the standard deviation of these experiments.

To begin modeling reactivation, we characterized the transcriptional response of reaerated MTB. Starting with the previously reported defined hypoxia model [18], [21], we transferred hypoxic cultures into roller bottles (5∶1 head space ratio, 15 rotations per minute) to promote rapid re-equilibration with atmospheric air, and growth at 37°C was assessed. Reaeration of MTB cultures was performed after 7 days of hypoxia, because earlier observations indicated that MTB transcription at 7 days closely resembles that at time points up to 3 weeks in this system (Rustad and Sherman, unpublished). Contrary to earlier reports [26], we found only very modest drops in viability following rapid shifts in oxygen tension (Figure 1).

#### Hypoxia-Repressed, Reaeration-Induced Genes

**Figure 2:**
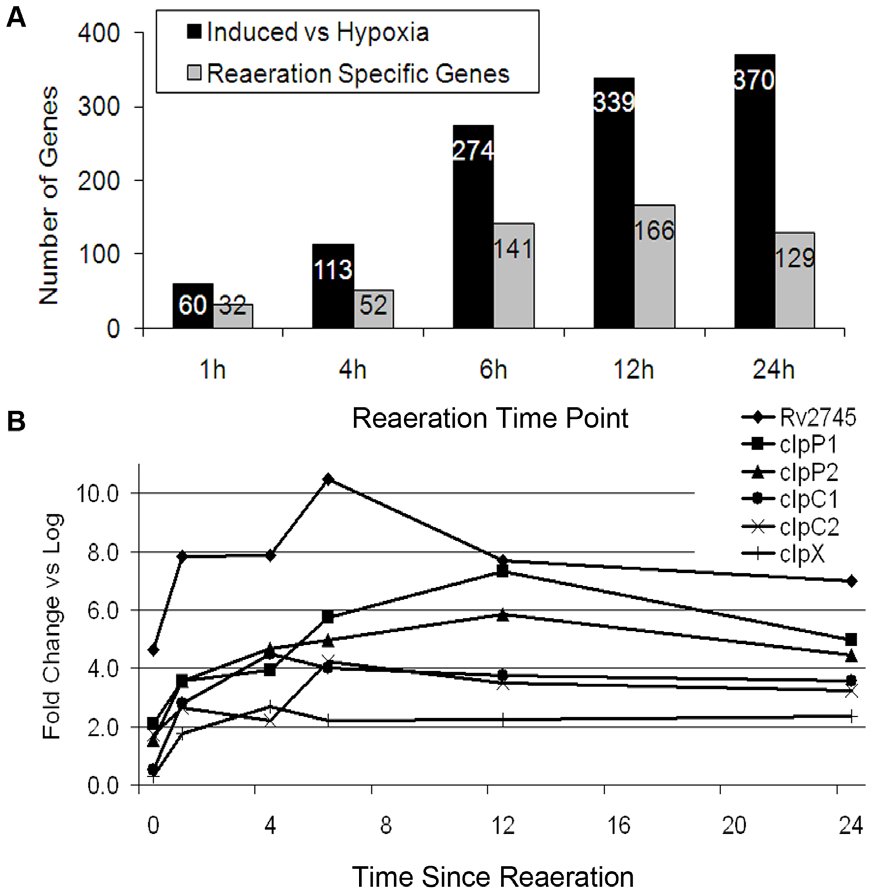
Transcription during reaeration of *M. tuberculosis*. A. Black bars are genes induced >2-fold compared to expression at 7 days hypoxia. Gray bars are the subset of these genes that are not repressed at 7 days hypoxia compared to log phase. Gray bars are defined as reaeration specific genes. B. Transcription of *clp* genes and Rv2745c during reaeration. Values represent fold change in gene expression compared to log phase, as measured by microarray and analyzed with Statistical Analysis of Microarrays on data from at least 2 biological replicates.

We hypothesized that reaerating MTB might express a distinct transcriptional response that could provide insight into the process by which MTB resumes replication following a period of bacteriostasis. Accordingly, we performed transcriptional profiling of reaerating MTB by whole genome microarray analysis. After 7 days hypoxia (time 0 for reaeration) and at subsequent times, samples were processed for microarray analysis as described [20], [21]. Reaeration induced a wave of transcription, which peaked at 24 hours with 370 genes induced at least two-fold (Figure 2A, Table 1 and Table S1). Not surprisingly, many induced genes are among those repressed during the transition from log phase to prolonged bacteriostasis [21] This transcriptional profile reflects the significant modifications MTB makes in order to survive hypoxia. MTB's return to a log phase transcriptional response represents the reversal of these bacteriostasis-associated adaptations. As cultures adapted to reaeration and neared replication, there was a progressive increase in the proportion of reaeration-induced genes that are originally hypoxia-repressed (Figure 2A).

**Table 1:** Genes induced at 6 hours and 12 hours of reaeration, in comparison to 7 days hypoxia (Num >1, q<0.5).

Rv #	Gene Description	Rv #	Gene Name
Rv0011c	conserved membrane protein	Rv2122c	phosphoribosyl-AMP pyrophosphatase hisE
Rv0076c	membrane protein	Rv2249c	glycerol-3-phosphate dehydrogenase glpD1
Rv0077c	oxidoreductase	Rv2359	ferric uptake regulation protein furB
Rv0140	conserved hypothetical protein	Rv2386c	isochorismate synthase mbtI
Rv0250c	conserved hypothetical protein	Rv2460c	ATP-dependent clp protease proteolytic subunit 2
Rv0251c	heat shock protein hsp	Rv2461c	ATP-dependent clp protease proteolytic subunit 1
Rv0276	conserved hypothetical protein	Rv2464c	DNA glycosylase
Rv0327c	cytochrome P450 135A1 cyp135A1	Rv2465c	ribose-5-phosphate isomerase
Rv0332	conserved hypothetical protein	Rv2504c	succinyl-CoA:3-ketoacid-CoA transferase subunit
Rv0350	chaperone protein dnaK	Rv2516c	hypothetical protein
Rv0351	chaperone grpE	Rv2643	arsenic-transport membrane protein arsC
Rv0352	chaperone protein dnaJ1	Rv2650c	phiRv2 phage protein
Rv0353	heat shock protein transcriptional repressor	Rv2672	secreted protease
Rv0383c	conserved secreted protein	Rv2674	methionine sulfoxide reductase
Rv0384c	endopeptidase ATP binding protein chain B clpB	Rv2690c	conserved alanine, valine and leucine rich membrane protein
Rv0416	hypothetical protein thiS	Rv2709	conserved membrane protein
Rv0563	protease transmembrane protein heat shock protein htpX	Rv2714	conserved alanine and leucine rich protein
Rv0586	transcriptional regulator, gntR-family	Rv2744c	conserved alanine rich protein
Rv0590A	MCE-family related protein	Rv2745c	transcriptional regulator
Rv0627	conserved hypothetical protein	Rv2865	conserved hypothetical protein
Rv0654	Dioxygenase	Rv2895c	mycobactin utilization protein viuB
Rv0753c	methylmalonate-semialdehyde dehydrogenase mmsA	Rv2913c	D-amino acid aminohydrolase
Rv0762c	conserved hypothetical protein	Rv3012c	glutamyl-tRNA(gln) amidotransferase subunit C gatC
Rv0790c	hypothetical protein	Rv3046c	conserved hypothetical protein
Rv0791c	conserved hypothetical protein	Rv3047c	hypothetical protein
Rv0793	conserved hypothetical protein	Rv3048c	ribonucleoside-diphosphate reductase beta chain
Rv0885	conserved hypothetical protein	Rv3054c	conserved hypothetical protein
Rv1013	polyketide synthase pks16	Rv3064c	conserved membrane protein
Rv1167c	transcriptional regulator	Rv3097c	triacylglycerol lipase, PE-PGRS family protein
Rv1168c	PPE family protein	Rv3160c	transcriptional regulator, tetR-family
Rv1218c	tetronasin-transport ATP-binding protein ABC transporter	Rv3164c	methanol dehydrogenase transcriptional regulator moxR3
Rv1222	conserved hypothetical protein	Rv3165c	hypothetical protein
Rv1224	hypothetical protein tatB	Rv3172c	hypothetical protein
Rv1234	transmembrane protein	Rv3177	Peroxidase
Rv1235	sugar-binding lipoprotein lpqY	Rv3188	conserved hypothetical protein
Rv1236	sugar-transport membrane protein ABC transporter sugA	Rv3189	conserved hypothetical protein
Rv1259	uracil dna glycosylase	Rv3201c	ATP-dependent DNA helicase
Rv1286	sulfate adenyltransferase/adenylylsulfate kinase	Rv3205c	conserved hypothetical protein
Rv1472	enoyl-CoA hydratase echA12	Rv3260c	transcriptional regulator whib-like whiB2
Rv1528c	polyketide synthase associated protein papA4	Rv3269	conserved hypothetical protein
Rv1590	conserved hypothetical protein	Rv3406	Dioxygenase
Rv1623c	membrane cytochrome D ubiquinol oxidase subunit I cydA	Rv3429	PPE family protein
Rv1673c	conserved hypothetical protein	Rv3515c	fatty-acid-CoA ligase fadD19
Rv1773c	transcriptional regulator	Rv3527	hypothetical protein
Rv1809	PPE family protein	Rv3530c	Oxidoreductase
Rv1817	Flavoprotein	Rv3531c	hypothetical protein
Rv1879	conserved hypothetical protein	Rv3538	Dehydrogenase
Rv1908c	catalase-peroxidase-peroxynitritase T katG	Rv3545c	cytochrome P450 125 cyp125
Rv1989c	hypothetical protein	Rv3839	conserved hypothetical protein
Rv2008c	conserved hypothetical protein	Rv3863	hypothetical alanine rich protein
Rv2053c	transmembrane protein	Rv3913	thioredoxin reductase trxB2
Rv2072c	precorrin-6y methyltransferase cobL		

Analysis of these genes indicates that during reaeration MTB induces the transcriptional and translational machinery, including synthesis of ribosomes, RNA polymerase, purines, pyrimidines, and amino acids (Table S1). MTB undergoes significant cell wall remodeling during the adaptation to bacteriostasis [27], and the reversal of this process is likely necessary in order for cell division to proceed. In this context, we note renewed transcription of the dTDP-rhamnose synthesis genes *rmlB* and *rmlC*, and peptidoglycan-synthesis genes including *murC, murF, murX,* and *ponA1.* Hypoxic repression and reaeration-induced transcription of genes for ATP synthase, cytochrome C oxidase and reductase, and NADH dehydrogenase suggest resumption of oxidative phosphorylation in reaerated MTB. Other genes that fit this transcriptional profile include the serine-threonine kinase *pknB*, virulence-associated *esxA* and *esxB*, and the cell division associated *wag31*. Consistent with the idea that expression of these hypoxia-repressed genes represents a return to log phase metabolism, our analysis shows that 28–50% of the hypoxia-repressed, reaeration-induced genes at any time point are predicted to be essential for growth of MTB *in vitro* [28].

#### Reaeration-Specific Genes

In addition to this progressive upregulation of log phase-associated genes, we also noted a subset of reaeration-induced genes that had not previously been repressed during hypoxia. These reaeration-specific genes, which we defined as those induced at least two-fold during reaeration relative to both late-stage hypoxia and log phase, were evident at the earliest time point (1 hour) and peaked by 12 hours. At these early times, reaeration-specific genes comprised about half of all induced loci (Figure 2A, gray bars).

There was substantial overlap in the particular reaeration-specific genes induced at each time point. To facilitate analysis of this transcriptional response, we defined the Reaeration Response as those reaeration-specific genes induced at both six and twelve hours, the two time points where the number of reaeration-specific genes is highest (Table 1). This list of 103 genes includes 8 transcription factors including the mce2 operon repressor *mce2R,* ferric uptake regulator *furB,* cell division regulator *whiB2, moxR3* and *Rv2745c*. Reaeration Response genes involved in metabolic adaptation include a sugar transporter (*Rv1235* and *Rv1236*) predicted to be essential for MTB survival in macrophages and mice [29]–[30], the triacylglycerol lipase *lipY* (*Rv3097c*), recently shown to cleave triacylglycerides upon reaeration, and a ribonucleotide reductase (*Rv3048c*) essential for aerobic growth [31]. The Reaeration Response also includes seven genes encoding protease or chaperone proteins, suggesting a need to degrade or refold proteins. Among these proteases are *clpP1, clpP2 and* their associated ATPase *clpC1.* Recent reports by Mehra et al. and Barik et al. have suggested that these Clp proteases may be involved in the response of *M. tuberculosis* to oxidative and surface stress, under the control of the putative transcription factor *Rv2745c* [32], [33]. These reports showed that transcript levels of *clpP1, clpP2 and clpC* were modulated by altered expression of *Rv2745c.* However, these studies did not address whether regulation of *clp* genes was directly controlled by *Rv2745c*. We sought to determine whether *Rv2745c* directly activated transcription of the MTB *clp* genes leading to higher Clp protease protein levels.

### Characterization of Transcriptional Regulator ClgR (Rv2745c)

The Rv2745c protein shows high homology (54–60% identity) with ClgR, a protein that activates transcription of Clp proteases in the related actinomycetes *Streptomyces coelicolor* and *Corynebacterium glutamicum.* As part of a self-regulation loop, ClgR both induces and is degraded by the Clp proteases in these organisms [34], [35], [36], [37]. As mentioned above, *Rv2745c* is part of the MTB Reaeration Response (Table 1). It is up-regulated in late hypoxia, and is further induced upon reaeration (Figure 2B). The two MTB Clp protease genes responsible for Clp protease substrate specificity, *clpC1, clpC2,* and *clpX,* are also up-regulated during reaeration (Figure 2B). Analysis of the promoter region of the *clpP1P2* operon reveals a perfect match with the *Corynebacterium* ClgR consensus binding site (WNNWMGCYNNNRGCGWWS) ninety-six base pairs upstream of the *ClpP1* start codon [36]. The *clpC1* promoter contains a copy of this consensus site with a single mismatch. Based upon the strong homology to ClgR, the consensus binding site and the expression pattern, we suspected that Rv2745c directly regulates *clp* protease gene expression in MTB. Recent reports have suggested that modulating transcription of *Rv2745c* or the *M. smegmatis* homologue affect transcript levels of the *clp* proteases [32], [33]; however, these studies did not assess whether or not this is a direct effect.

**Figure 3:**
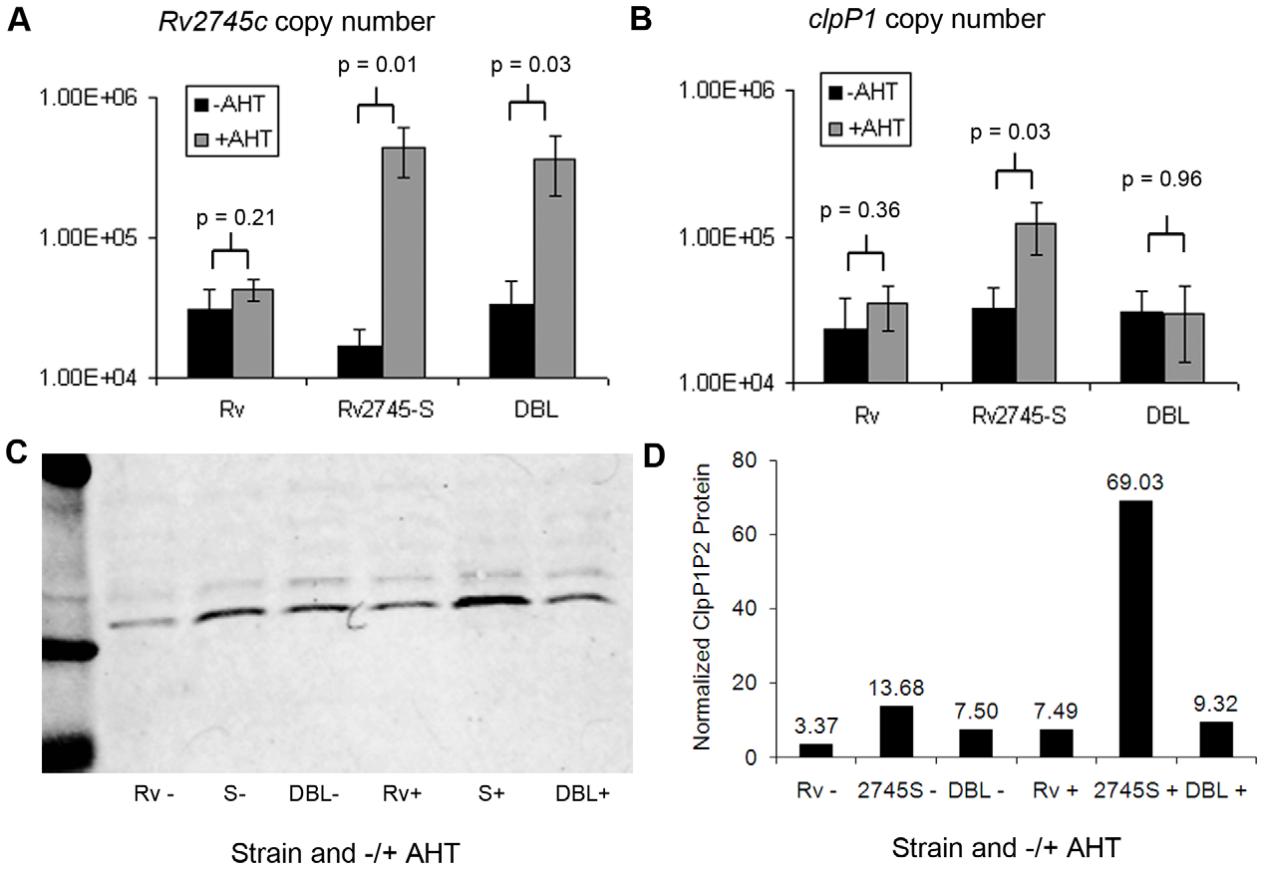
Rv2745c activates transcription of the *clpP1P2* operon. A and B. Shown are the mRNA copy number of Rv2745c and clpP1P2 in the presence and absence of anhydrotetracycline (AHT), normalized to *sigA*. Data are three technical replicates, representative of three biological replicates. p values indicate results of a two tailed unpaired t test. S  =  Rv2745-S, DBL  =  L24SR25D. C. Western blot of ClpP1P2 expression. D. Quantification of ClpP1 and ClpP2 Western blot band intensity normalized to Protein Tyrosine Phosphatase B band intensity from same samples. Data are representative of three independent experiments.

To test whether Rv2745c regulates MTB *clp* genes, we employed a tetracycline-inducible overexpression system. However, as noted above ClgR is a Clp substrate in other bacteria. In *Streptomyces*, cleavage depends upon the ClgR C-terminus, which bears strong similarity to the MTB Rv2745c C-terminus. Since induced degradation of the regulator would confound results, we generated an altered *Rv2745c* (*Rv2745-S,* for stabilized) in which the final two amino acids (V111, A112) were mutated to aspartates. This mutation prevents degradation of ClgR by the Clp proteases in *Streptomyces* [35]. In the presence of anhydrotetracycline (AHT), *Rv2745-S* mRNA copy number increased 25-fold compared to the strain without AHT (Figure 3A). We used this inducible system to assess whether overexpression of *Rv2745-S* resulted in increased *clpP1P2* transcription. In the presence of excess *Rv2745-S* mRNA, ∼4-fold more *clpP1P2* mRNA was detected, consistent with the expression level of these genes during reaeration (Figures 2B and 3B). Furthermore, levels of the ClpP1 and ClpP2 proteins in MTB were also increased by *Rv2745-S* overexpression (Figure 3C and D, ClpP antibody recognizes both subunits).

**Figure 4:**
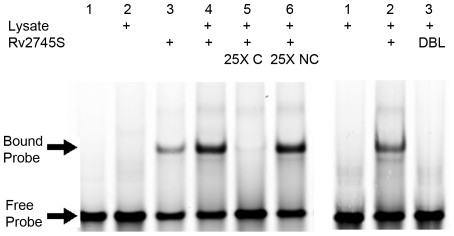
Specific binding of Rv2745c to the *clpC1* promoter and disruption of DNA binding by targeted Rv2745c mutation. Binding reactions were performed with 200pmol of the MTB clpC1 promoter fragment as a probe. Lysate  =  MTB protein lysate; C  =  unlabeled probe competitor; NC  =  unlabeled nonspecific DNA; DBL  =  L24SR25D mutant form of Rv2745S.

We assessed direct interaction of the Rv2745c protein with the *clpC1* and *clpP1P2* promoters. Due to insolubility of the wild-type protein, we used the Rv2745-S described above. Purified Rv2745-S protein was incubated with DNA in an electrophoretic mobility shift assay (EMSA). A shift of the *clpC1* promoter was observed in the presence of Rv2745-S, and DNA-binding could be out-competed by 25-fold excess unlabeled probe, but not by a non-specific competitor (Figure 4). Similar results were obtained with the *clpP1P2* promoter fragment (data not shown). To assess if overexpression of MTB *clpP1P2* is really due to transcriptional activation by Rv2745-S, we sought to interrupt the DNA binding activity of this protein. The Rv2745c sequence predicts a helix-turn-helix DNA-binding motif [34], [38], and by overlaying the Rv2745c protein sequence on a recently published structure of the *Corynebacterium glutamicum* ClgR protein [38], [39], [40] we identified residues with predicted direct DNA interactions. We used site-directed mutagenesis to create proteins with alterations in the putative helix-turn-helix region, R25D, R43D and L24SR25D, and assessed the ability of these mutant proteins to act as transcription factors. Overexpression of these mutant proteins occurred on addition of anhydro-tetracycline (Figure 3A, L245SR25D data shown are representative of results for all three mutants), but a concomitant increase in transcription of *clpP1P2* was not observed (Figures 3B and C). In addition, EMSA analysis demonstrated that the mutants failed to bind *clpC1* and *clpP1P2* promoter DNA (Figure 4 and data not shown). From these results, as well as the high degree of homology to *clgR* in related organisms, we conclude that *Rv2745c* is the MTB *clgR* orthologue.

## Discussion

While the MTB adaptations to hypoxic bacteriostasis have received considerable attention, little is known about the mechanism by which bacilli reactivate. Early studies suggested that growth-arrested MTB responds to re-oxygenation with immediate RNA synthesis, while DNA synthesis and replication are delayed [10], [14]. More recent work revealed that triacylglycerols stored during hypoxia are cleaved during reaeration of *Mycobacterium bovis* bacillus Calmette-Guerin by *lipY* (*Rv3047c)* and other lipases [41]. The resuscitation promoting factors (RPFs) encoded by MTB also appear to have a role in chronic infection and reactivation in a mouse model [42], [43], [44], [45].

To gain more mechanistic insights, we developed a model in which MTB resumes replication following reaeration under defined conditions *in vitro*. Replication is preceded by two distinct transcriptional phases. The latter of these, steadily building in number as MTB nears replication, includes genes involved in transcription, translation, oxidative phosphorylation and fatty acid synthesis (Table S1). Since genes involved in each of these functions are repressed as MTB adapts to hypoxia [21], the bacillus would be expected to resume their expression while adapting to reaeration and preparing for log phase growth. Notably, approximately one-third of the genes in this late pre-replication transcriptional profile are predicted to be essential for MTB survival *in* vitro [28]. The increased transcription we observed for genes involved in purine and pyrimidine synthesis, RNA polymerase, amino acids synthesis, and ribosome components agrees with previous estimates that levels of protein, RNA and ribosomes increase in actively growing as compared to slower growing bacilli [46]. In addition, it was shown recently that MTB adapted to a non-replicating state in response to hypoxia or nutrient deprivation has a substantially lower ATP content than replicating MTB [16], [47]. These data suggest that metabolically slowed MTB synthesize fewer of these crucial components of transcription and translation, possibly due to energy limitations. Perhaps during bacteriostasis, these adaptations are part of a strategy to conserve energy for survival.

The earlier transcriptional adaptation we observed, the Reaeration Response, is a set of ∼100 genes induced when compared to both long-term hypoxia and log-phase (Figure 2A and Table 1). We hypothesized that it would be necessary for MTB to undergo metabolic changes prior to replication and that an identifiable transcriptional modulation would characterize this shift. Consistent with this possibility, the Reaeration Response includes several genes involved in reordering bacterial metabolism, including a ribonucleotide reductase gene essential for aerobic growth along with genes involved in fatty acid metabolism, sugar transport, and a triacylglycerol lipase (*lipY*), which may facilitate utilization of stored triacylglycerols during reaeration [31], [41]. In addition, enrichment of chaperone proteins and proteases in the Reaeration Response suggests an effort by MTB to repair or replace proteins and cellular components damaged by either hypoxia and/or re-introduction of oxygen. Within the Reaeration Response, we note a two- to ten-fold enrichment of genes upregulated in response to other stresses including diamide, heat shock, acid stress, and macrophage infection [48], [49], [50], [51], suggesting that MTB senses and responds to stress during reaeration.

The Reaeration Response also includes eight putative transcription factors. One of these is Rv2745c, a protein similar to the ClgR regulators of related actinomycetes [36], [37], [52]. In these species, ClgR binds to a conserved sequence upstream of *clpP* and activates transcription of this gene. We demonstrate that Rv2745c is a ClgR homologue that directly activates transcription of Clp proteases in MTB. While this work was ongoing, two independent studies reported modulation of *clpP* gene transcription upon alteration of *Rv2745c* mRNA levels [32], [33]. The first study demonstrated that inducible overexpression of *Rv2745c* led to higher transcript levels of *clpP1, clpP2* and *clpC1* [33]. The second study showed that depletion of the *Mycobacterium smegmatis* homologue of *Rv2745c* by antisense prevented upregulation of *clpP1,clpP2,* and *clpC1* transcription upon exposure to vancomycin [32]. Our data confirm and extend these findings, showing that ClpP protein levels as well as transcript levels increase with *Rv2745c* overexpression (Figure 3C and D), and also that Rv2745c directly interacts with the *clpC1* and *clpP1P2* promoters (Figure 4 and data not shown). Interestingly, *Rv2745c* transcription in response to diamide stress is dependent on the sigma factor *sigH* [33], which we previously have shown is upregulated during extended hypoxia [21]. These findings suggest shared mechanisms in MTB responses to stress conditions likely to be relevant *in vivo*. It will be interesting to decipher which other components of the MTB Reaeration Response are shared with other stress responses, and which are specific to increased oxygenation.

Clp proteases and their regulators play crucial roles in maintaining and overcoming replication checkpoints in other bacteria. For example, the *Caulobacter crescentus* regulator CtrA both transcribes a set of genes involved in replication and binds to the chromosomal origin, silencing initiation of replication, and degradation of CtrA by the Clp proteases is needed for replication to begin [53]. In *S. lividans*, ClgR overexpression delays a morphological transition that the Clp proteases accelerate, and stabilizing ClgR to prevent its degradation intensifies this effect [37], [54].

The set of genes regulated by ClgR is not yet fully characterized. A pattern search for the conserved ClgR binding motif within MTB shows matches upstream of *cysK1, Rv2224c, Rv0037c, hemD, and PPE55*. Allowing a single mismatch yielded a list of 101 genes with a putative ClgR binding site, including the ATPase *clpC1 and clgR* itself. The transcriptional patterns of these genes during reaeration are varied, suggesting that this process is mediated by more than one regulator. Seven other transcription factors were identified in the Reaeration Response, and their regulons remain to be determined.

These studies introduce a model for probing the mechanisms of reactivation of MTB *in vitro.* Here, we describe the transcriptional response of MTB to reaeration, and identify the Clp proteases and their transcription factor ClgR as upregulated during the transition of MTB from bacteriostasis to growth. Further exploration of this model may reveal other players in MTB's adaptation to an active, replicating state, and it will be interesting to assess the extent to which induction of the Reaeration Response occurs in other strains, including clinical isolates. In addition, while the adaptations of MTB to hypoxic/reaerated microenvironments are likely central to the pathogen's success, the *in vivo* relevance of the Reaeration Response remains to be determined.

Determining the means by which the bacterium adapts to these changing conditions will open doors to potential new drug therapies and diagnostics. The roles of protein degradation and refolding in MTB stress response are just beginning to be revealed. Recently, the MTB proteasome was shown to have a role in adaptation to nitrosative stress, and compounds were identified which kill MTB by selectively targeting the MTB proteasome over the human counterpart [55], [56]. The Clp proteases might prove a similarly viable new drug target. Additionally, genes of the Reaeration Response could contribute to the development of new and effective diagnostics. Identifying which latently-infected individuals are most likely to suffer from reactivation of TB is an important priority in TB research [57]. Further studies are needed to explore whether immunological recognition of Reaeration Response proteins correlates with the likelihood of reactivation among MTB-infected individuals.

## Supporting Information

Table S1 Hypoxia-repressed genes that are induced during M. tuberculosis reaeration.
(0.08 MB XLS)
